# The impact of gastric pouch size, based on the number of staplers, on the short-term weight outcomes of Roux-en-Y gastric bypass

**DOI:** 10.1016/j.amsu.2022.104914

**Published:** 2022-11-18

**Authors:** Neda Haghighat, Hooman Kamran, Mohammad Naser Moaddeli, Babak Hosseini, Ali Karimi, Iman Hesameddini, Masoud Amini, Seyed Vahid Hosseini, Abtin Vahidi, Nader Moeinvaziri

**Affiliations:** a Laparoscopy Research Center, Shiraz University of Medical Sciences, Shiraz, Iran; b Colorectal Research Center, Shiraz University of Medical Sciences, Shiraz, Iran

**Keywords:** Bariatric surgery, Pouch, Gastric bypass, Weight loss, Surgical staplers

## Abstract

**Introduction:**

No standard of anatomical variables, including stoma size, limb length, pouch size, and volume, has been determined for laparoscopic Roux-en-Y gastric bypass yet. Herein, we evaluated the effect of two different techniques for creating the gastric pouch on short-term postoperative weight loss.

**Methods:**

This retrospective cohort was conducted on patients with a laparoscopic Roux-en-Y gastric bypass history from January 2019 to September 2020. Patients were divided into two groups: in one group, patients’ gastric pouch was made using two 60 mm linear staplers, while in the other group, the gastric pouch was made using three 60 mm linear staplers. Anthropometric data, including weight, height, and body mass index (BMI), were measured preoperatively and six months following surgery. Weight outcomes, such as weight loss, a decrease in BMI, excess weight loss (%EWL), and total weight loss (%TWL), were calculated as short-term weight outcomes.

**Results:**

Two groups, each containing 50 patients, were included. Patients with smaller pouches (two staplers) had 32.4 ± 9.2 kg weight loss, and those with larger pouches (three staplers) had a 31.42 ± 10.3 kg weight loss. Also, %EWL was 69.7 ± 14.9 and 63.0 ± 20.9, and %TWL was 28.2 ± 6.0 and 26.14 ± 7.5 in patients with two stapler pouches and three stapler pouches, respectively. None of the weight outcome parameters were significantly different between the groups (p-value>0.05).

**Conclusion:**

Various studies have been conducted, resulting in different conclusions regarding the effect of the size of the gastric pouch on weight loss. One of the major differences contributing to varying literature studies results is the measurement method used for gastric pouch size. We conclude that using two staplers is not a way to achieve a better result. As the best measurement method has not been defined, studies comparing different methods are suggested; here, the aim was to use a more simple and clinical method regarding this issue.

## Highlights

• The historical consideration of a small gastric pouch for better weight results.
• Incompatibility of study results due to different measurements for pouch size.
• No significant differences in weight outcomes between two and three staplers.
• The need for further high-quality studies to determine the best measurement method.

## 1 Introduction

The prevalence of obesity has been steadily increasing in recent decades and has now become a public health problem [1]. Bariatric surgery is known as the most effective method of treating morbidly severe obesity [[2], [3], [4]]. Nowadays, one of the most common bariatric surgeries preferred, especially in diabetic patients, is laparoscopic Roux-en-Y gastric bypass [5,6]. Several factors contributed to the lack of ideal results in postoperative weight loss and weight regain, some of which are generally related to the patients’ characteristics, including male gender, age, greater initial weight, the coexistence of diabetes mellitus, and some of which are related to surgery [[7], [8], [9]].

Unfortunately, there is still no standard for anatomical variables, such as stoma size, limb length, pouch size, and volume [9,10]. So far, many studies have been done on pouch size and its effect on weight loss, which have often been descriptive and observational [[11], [12], [13]]. In a 2011 study at Cleveland Clinic, 71% of patients who underwent endoscopic examination due to weight regain had enlarged pouch and/or stoma size [14]. Another study conducted in 2017 in Brazil showed that smaller pouches led to faster gastric emptying, more significant weight loss maintenance, and better food tolerance [15]. On the other hand, many studies, including a systematic review conducted in 2020, have shown no correlation between pouch size and postoperative weight loss [16].

In addition, some efforts have been made to achieve a standard for pouch size. A study conducted in the Netherland in 2018 showed that using the calibration tube for gastric pouch creation may result in higher excess weight loss (%EWL) and total weight loss (%TWL) at two years of follow-up [17]. Also, most studies only emphasize the smaller size of the pouch, but a 2020 study in the Netherland found that an extended pouch (pouch length 10 cm) can lead to reduced weight regain compared to a pouch length of 5 cm [18].

In this study, we evaluated the short-term effect of two techniques for creating the gastric pouch in laparoscopic Roux-en-Y gastric bypass on %EWL and %TWL. We hypothesized that using only two staplers to create the gastric pouch may lead to higher %EWL and %TWL than three staplers.

## 2 Methods

The study was designed as a retrospective cohort conducted on patients with a history of laparoscopic Roux-en-Y gastric bypass in Mother and Child hospital, affiliated with Shiraz University of Medical Sciences, Shiraz, Iran, from January 2019 to September 2020. Adult patients aged 18–65 years were considered for bariatric surgery if they had body mass index (BMI) > 40 kg/m2 or >35kg/m2 with obesity-related comorbidity. If indicated, all patients were preoperatively evaluated by a multidisciplinary team consisting of dietitians, bariatric surgeons, endocrinologists, and psychologists. Patients with a history of smoking, alcohol use, psychiatric problems, inflammatory and infectious diseases, cancer, and heart and/or lung failure were excluded. Moreover, patients with reversal or previous bariatric surgery were excluded from the study.

**Fig. 1:**
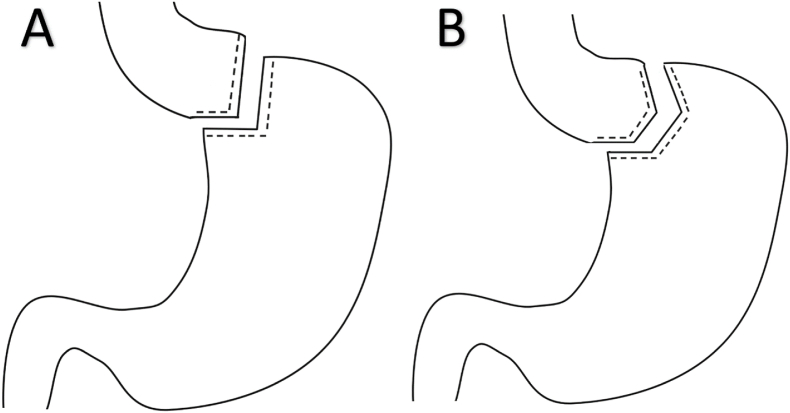
Scheme for both techniques of Roux-en-Y gastric bypass; A) two-stapler and B) three-stapler.

All patients underwent classic Roux-en-Y gastric bypass with the standard method [19,20] and the same technique. Patients were divided into two groups based on variation in the steps of gastric pouch creation. After the creation of the pneumoperitoneum, four ports were used for both procedures. In one group, the patients’ gastric pouch was made using two 60 mm linear staplers, one horizontally and another vertically. In contrast, in the other group, the gastric pouch was made using three 60 mm linear staplers, one horizontally and two vertically (Fig. 1). The following steps of the surgery were performed similarly for both procedures. The jejunum was divided 100 cm from the ligament of Treitz, and a 3 cm gastrojejunostomy was created. The alimentary limb was also 100 cm.

Informed consent was obtained from all individual participants included in the study. The study protocol conforms to the provisions of the Declaration of Helsinki, and the Shiraz University of Medical Sciences Ethics Committee approved this study protocol based on the code of IR.SUMS.MED.REC.1400.344. The work has been reported in line with the STROCSS criteria [21].

### 2.1 Measures and outcomes

Anthropometric data, including weight, height, and BMI, were carefully measured at preoperative and six-month postoperative medical visits. Using these data, the percentages of TWL and EWL that are the primary outcomes of this study were calculated. The percentage of TWL was defined as weight loss divided by weight before surgery, and the percentage of EWL was defined as weight loss divided by excess weight before surgery above a normal BMI of 25 kg/m2. The patients' age, sex, exercise activities, and underlying diseases were also recorded.

### 2.2 Statistical analysis

All data were expressed as mean ± standard deviation and were analyzed using SPSS software, version 17. The Kolmogorov-Smirnov test was used to follow the data from the normal distribution. Chi-square was done for nominal variables, and an independent *t*-test was used to compare the mean changes of quantitative variables. The Mann-Whitney test was used to compare variables that did not have a normal distribution. P-value<0.005 was considered statistically significant.

## 3 Results

### 3.1 Demographic and preoperative data

A total of 100 patients were included in the study. The mean age of patients was 35.8 ± 10.1 years. In the group of patients with a smaller pouch, which was made by using two 60 mm staplers, the mean age was 35.7 ± 9.1 years, and in the group of patients with a larger pouch, which made by using three 60 mm staplers, the mean age was 35.9 ± 11.1. In the former group, 20% of patients were male with a mean BMI of 42.4 ± 3.4; in the latter group, 28% were male with a mean BMI of 46.4 ± 22.1. Demographic and preoperative clinical data of patients are shown in Table 1. As demonstrated, except for initial weight, other preoperative data were not significantly different between the groups (p-value>0.05).

**Table 1:** Demographic and preoperative clinical data of patients.

Variable	Two staplers (n = 50)	Three staplers (n = 50)	P-value
Age, year; mean (SD)	35.72 (9.12)	35.90 (11.12)	0.909
Gender; n (%)			
Male	10 (20%)	14 (28%)	0.349
Female	40 (80%)	36 (72%)	
Weight, kg; mean (SD)	114.28 (15.63)	119.52 (14.00)	**0.029**
BMI, kg/m2; mean (SD)	42.44 (3.45)	46.42 (22.12)	0.164
Height. cm; mean (SD)	163.86 (8.59)	164.24 (14.18)	0.109

SD: standard deviation, BMI: body mass index.

### 3.2 Weight loss outcomes

**Fig. 2:**
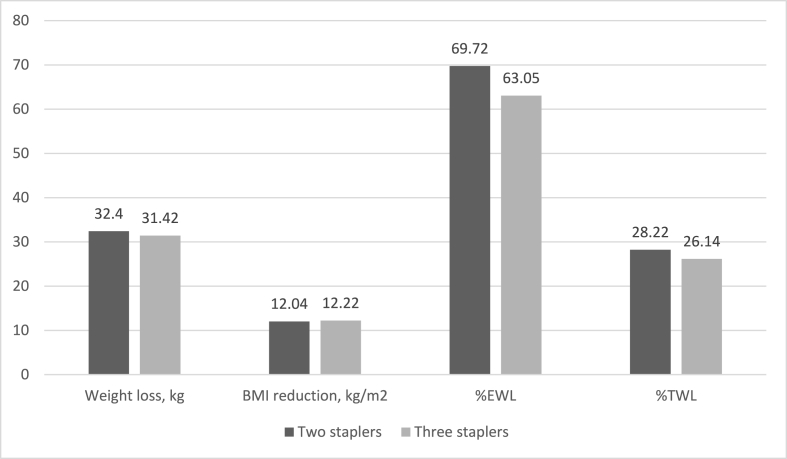
The comparison of weight loss, BMI reduction, %EWL, and %TWL between the two-stapler and three-stapler groups; none of the differences were statistically significant (p-value>0.05). BMI; body mass index, %EWL: excess weight loss, %TWL: total weight loss.

Patients with smaller pouches had 32.4 ± 9.2 kg weight loss after six months, with a 12.0 ± 3.2 decrease in their BMI. The group of patients with pouches made using three staplers had 31.42 ± 10.3 kg weight loss and a 12.2 ± 7.3 decrease in their BMI after six months. %EWL in patients with two stapler pouches and three stapler pouches were 69.7 ± 14.9 and 63.0 ± 20.9, respectively. The %EWL in the two-stapler and three-stapler groups were 32.31–111.29 and 31.25–108.70, respectively. Besides, four (8%) in the two-stapler group and 16 (32%) in the three-stapler group had an %EWL lower than 50%. As shown in Table 2, none of the parameters were significantly different between the groups (p-value>0.05) (Fig. 2).

**Table 2:** Weight loss outcomes six months following operation between two-stapler and three-stapler groups.

Variable	Two staplers (n = 50)	Three staplers (n = 50)	P-value
Weight loss, kg; mean (SD)	32.40 (9.23)	31.42 (10.30)	0.804
BMI decrease, kg/m2; mean (SD)	12.04 (3.20)	12.22 (7.30)	0.374
EWL, %; mean (SD)	69.72 (14.95)	63.05 (20.93)	0.070
TWL, %; mean (SD)	28.22 (6.05)	26.14 (7.57)	0.133

SD: standard deviation, BMI: body mass index, EWL: excess weight loss, TWL: total weight loss.

## 4 Discussion

The size of the gastric pouch has been postulated to be an influential variable for gastric bypass outcomes in such a way that a small pouch was considered an important factor for better weight outcomes [22]. Although different methods for measuring gastric pouch size have been utilized to evaluate this issue, the best method has not been determined yet. These studies also had different conclusions, which may be attributable to the measurement methods applied. In this study, to decrease the measurement error, gastric pouch size was determined by the number of staplers used in Roux-en-Y gastric bypass for creating the gastric pouch. We only had two groups (two-stapler and three-stapler), each containing 50 patients. Herein, the weight outcome was not associated with pouch size.

In a study by Nishie et al. [23], authors included 82 cases, evaluating gastric pouch size correlation with short-term weight loss following laparoscopic Roux-en-Y gastric bypass. In their study, pouch size measurement was done by upper gastrointestinal contrast study, which showed no correlation between the size and %EWL at 3, 6, 12, and 24 months following the operation. Similarly, we did not observe any significant association between pouch size and weight outcome after six months from the surgery; we used weight loss, BMI decrease, %TWL, and %EWL for measuring weight outcomes, which none of them were statistically associated with the number of staplers (p-values = 0.804, 0.374, 0.133, and 0.070, respectively). But on the other hand, a study by Campos et al. [8], which used swallow studies for calculating pouch area, had the opposite result. In their research, the pouch area had an inverse relationship with %EWL, meaning poorer outcomes in cases with larger pouch areas.

A systematic review in 2020 gathered studies that evaluated the gastric pouch or gastrojejunostomy size on weight outcomes following Roux-en-Y gastric bypass; of fourteen studies found, five reported poorer results for larger pouches [8,9,[24], [25], [26]], while other studies did not find any associations between weight outcome and pouch size [[11], [12], [13],^15^,^23^,[27], [28], [29], [30]]. The mentioned review article concluded that larger pouches were associated with poorer outcomes. However, they stated that due to the poor quality of studies and different measurement techniques, drawing a definite conclusion was difficult, and further investigations were suggested [16].

One of the most important factors contributing to these conflicting results is the measurement technique used, and an optimal method has not been introduced yet. Studies have used various techniques in the literature, including contrast study, 3D computed tomography scan (CT), barium swallow, and even cottage cheese test [11,13,29,30]. In the current study, we used the number of staplers as a tool for the pouch size measurement, and cases were divided into two groups of two-stapler and three-stapler. The number of staplers fired has been used in some other studies to measure the gastric pouch [12]; however, in our research, we only had two groups of patients that were compared. We used this method to reduce the measurement error and get a more reliable result.

The pouch sizes in our study were based on the number of staplers, and it is conceivable that the two-stapler group was not small enough to affect the average change in weight loss significantly. Therefore, studies with an accurate method for measuring of gastric pouch size are suggested. Otherwise, malabsorption or hormonal changes rather than mechanical restriction could be more effective regarding weight outcome [23].

Our study was not without limitations. This was a retrospective study with its limitations compared to the prospective ones. Although we aimed to assess the short-term weight outcomes in this study, follow-up of more than six months is suggested for further studies showing the results in the long term. We had two groups, each containing fifty cases; although the sample size was not small, larger sample sizes are suggested for more reliable results. As the measurement of choice for gastric pouch size is unknown and different conclusions have been achieved in previous studies, more high-quality studies are needed to determine the best measurement method.

## 5 Conclusion

The small gastric pouch has been historically considered an important factor for better weight outcomes. Various studies have been conducted to evaluate this issue, resulting in different conclusions. One of the major differences contributing to the studies’ results is the measurement method used for gastric pouch size. We used two different procedures as a clinical method of measurement. And our hypothesis regarding higher %EWL and %TWL in the two-stapler procedure was not supported based on our outcomes. Therefore, the result of a number of studies showing poorer weight outcomes with larger pouches is questioned. Here, we conclude that using two staplers is not a way to achieve better weight loss. As the best measurement method has not been defined, studies comparing different methods are suggested; in this study, the aim was to use a more simple and more clinical method for this issue.

## Ethical approval

The University Ethics Committee approved this study protocol based on the code of IR.SUMS.MED.REC.1400.344.

## Sources of funding

The present study was supported by a grant from the Vice-chancellor for Research, 10.13039/501100004320Shiraz University of Medical Sciences, Iran.

## Author contribution

Neda Haghighat: Data collection and interpretation, writing and revising the paper.

Hooman Kamran: Data analysis, writing the paper.

Mohammad Naser Moaddeli: Data gathering, revising the paper.

Babak Hosseini: Data interpretation, revising the paper.

Ali Karimi: Data interpretation, revising the paper.

Iman Hesameddini: Data collection, writing the paper.

Masoud Amini: Data interpretation, revising the paper.

Seyed Vahid Hosseini: Supervising, revising the paper.

Abtin Vahidi: Data interpretation, writing the paper.

Nader Moeinvaziri: Study concept and design, data interpretation, revising the paper.

## Registration of research studies

1. Name of the registry: Vice-chancellor for Research, Shiraz University of Medical Sciences, Iran
2. Unique Identifying number or registration ID: IR.SUMS.MED.REC.1400.344
3. Hyperlink to your specific registration (must be publicly accessible and will be checked): https://ethics.research.ac.ir/ProposalCertificateEn.php?id=226325&Print=true&NoPrintHeader=true&NoPrintFooter=true&NoPrintPageBorder=true&LetterPrint=true

## Guarantor

Nader Moeinvaziri, MD.

## Consent

Informed consent was obtained from all individual participants included in the study.

## Provenance and peer review

Not commissioned, externally peer-reviewed.

## Declaration of competing interest

None declared.
